# Comparison of the Efficacy of Tap Water Iontophoresis Versus Aluminum Chloride Hexahydrate in the Treatment of Palmoplantar Hyperhidrosis

**DOI:** 10.7759/cureus.32367

**Published:** 2022-12-09

**Authors:** Muhammad Rahim, Najia Ahmed, Kiran Naz Khan, Shafia Memon, Tehseen Naveed, Syed Arbab Shah, Omer Farooq, Usman Ali

**Affiliations:** 1 Department of Dermatology, Bahria University of Health Sciences, Pakistan Navy Station (PNS) Shifa Hospital, Karachi, PAK; 2 Department of Dermatology, National University of Medical Sciences, Combined Military Hospital (CMH) Peshawar, Peshawar, PAK; 3 Department of Ophthalmology, Bahria University of Health Sciences, Pakistan Navy Station (PNS) Shifa Hospital, Karachi, PAK; 4 Department of Oncology, National University of Medical Sciences, Combined Military Hospital (CMH) Rawalpindi, Rawalpindi, PAK

**Keywords:** palmoplantar hyperhidrosis, hdss, treatment, palmoplantar, starch iodine test, aluminum chloride, iontophoresis, hyperhidrosis, efficacy

## Abstract

Objective: To compare the efficacy of tap water iontophoresis (TWI) versus aluminum chloride (AC) hexahydrate in the treatment of palmoplantar hyperhidrosis.

Methods: The study was a randomized control trial performed at the dermatology department of Pakistan Navy Station (PNS) Shifa Hospital, Karachi from March 2022 to September 2022. A total of 70 palmoplantar hyperhidrosis patients were included in the study after getting approval from the ethical committee. Patients were divided into two groups. Group A patients were treated with TWI three times a week for four weeks. Group B patients were treated with a 20% AC topical solution applied at night to the affected areas for four weeks. The Hyperhidrosis Disease Severity Scale (HDSS) score for both groups was calculated at baseline, one, two, three, and four weeks. The final response was labeled at four weeks by comparing mean HDSS reduction in both groups. SPSS version 28 (IBM Corp., Armonk, NY) was used for data analysis.

Results: Mean HDSS was compared for both groups at the end of the study, which showed a significant reduction in the mean score from 3.40 ± 0.65 to 1.48 ± 0.78 in group A, as compared to a decline in scores in group B from 3.28 ± 0.67 to 2.14 ± 0.94 (p = 0.002). In group A, zero, one, two, and three points HDSS improvement was 2.9%, 25.7%, 48.6%, and 22.9%, respectively. Whereas in group B, it was 34.3%, 22.9%, 34.3%, and 8.6%, respectively (p = 0.001).

Conclusion: As compared to AC topical solution, TWI is an effective, safe, and inexpensive management option for palmoplantar hyperhidrosis. It causes more improvement in HDSS scores and has lesser side effects.

## Introduction

Hyperhidrosis is the increased generation of sweat above what is physiologically required for thermal equilibrium. This disorder is classified as primary (idiopathic) and secondary [1]. Primary hyperhidrosis is not due to an outside stimulus, body temperature, or an underlying disease. The hypothesized mechanism of primary hyperhidrosis is the combined effect of both reduced threshold and enhanced sweating response [2]. It affects approximately 2-3% of the general population [3]. Secondary hyperhidrosis is a less common condition with an underlying cause including infections, endocrine disorders, or adverse effects of medications [4]. Hyperhidrosis significantly impairs the patient’s daily activities, social life, and occupation, leading to anxiety and depression [5,6]. Impairment of quality of life (QOL) is more than in other cutaneous conditions like acne, eczema, or psoriasis [7]. A variety of management options have been used to control or reduce the excessive sweating involving the palms, soles, and axilla. These include lifestyle and behavioral changes, topical antiperspirants like aluminum chloride (AC), tap water iontophoresis (TWI), botulinum toxin injections, systemic anticholinergic medications, energy-delivering devices, surgery, and sympathectomy [8]. AC is used as first-line therapy for primary hyperhidrosis. It is proposed that the metal ions in the aluminum salts are responsible for damaging epithelial cells with sweat gland ducts, resulting in plug formation leading to obstruction of these ducts and decreased sweating [9]. Iontophoresis is used as a second-line treatment for primary hyperhidrosis. Direct electrical current is passed through the skin in tap water allowing movement of ions across the skin surface. It is postulated that an increase in hydrogen ions decreases the pH of the skin, resulting in blockage of ducts or causing disturbance of sweat gland sympathetic nervous transmission [10].

Since palmoplantar hyperhidrosis disrupts the patient’s routine activities and there is currently no such treatment that can completely treat hyperhidrosis, it causes considerable psychosocial distress. Therefore, our study aims to compare the efficacy and side effects of TWI with AC. To the best of the available literature, there is no direct comparison between TWI and AC in the management of palmoplantar hyperhidrosis and there is also no registered randomized controlled trial (RCT) on the comparison of TWI and AC. TWI, if found successful, can be recommended as an effective treatment for palmoplantar hyperhidrosis as it is safe, easily available, and affordable for the patient.

## Materials and methods

This RCT was carried out from March 2022 to September 2022 in the dermatology department of Pakistan Navy Station (PNS) Shifa Hospital, Karachi, after obtaining ethical committee approval (Reference No.: ERC/2021/DERMA/49). The study was registered with the Australian New Zealand Clinical Trials Registry (ACTRN1622000324718). A total of 70 palmoplantar hyperhidrosis patients were included in the study. After explaining the study, an informed consent form was signed by all the patients.

Inclusion criteria

Patients aged 10 to 50 years, both males and females, with hyperhidrosis of at least two months duration and the Hyperhidrosis Disease Severity Scale (HDSS) score of 2, 3, or 4 at presentation were included.

Exclusion criteria

Patients who had undergone surgical treatment, botulinum toxin treatment for palmoplantar hyperhidrosis, or were suffering from organic disorder resulting in hyperhidrosis like hyperthyroidism were excluded from the study.

Patients were randomly divided into two groups by using sealed opaque envelopes: group A (35 patients) and group B (35 patients). The order in which the subjects were randomized was determined by coin tossing. The people assessing the outcome were blinded. During the first visit, after a complete history and detailed examination, HDSS was calculated for each patient and a starch iodine test was done to define areas and localize hyperhidrosis. HDSS is a four-point scale that includes the following points: (1) my sweating is never noticeable and never interferes with my daily activities; (2) my sweating is tolerable but sometimes interferes with my daily activities; (3) my sweating is barely tolerable and frequently interferes with my daily activities; (4) my sweating is intolerable and always interferes with my daily activities [5].

In group A patients, TWI treatment was used for both hands and feet. An electric current (10-20 mA) was delivered across the skin by using simple tap water. Hands and feet were submerged in the TWI machine for 10 minutes. These sessions were done three times a week for four weeks. Group B patients were treated with a 20% AC topical solution. It was applied topically to hands and feet (5-10 mL) at night for four weeks. HDSS was calculated at presentation and then every week for four weeks after the initiation of treatment. The final response was assessed at four weeks.

Data were analyzed by Statistical Package for the Social Sciences (SPSS) version 28 (IBM Corp., Armonk, NY). Frequencies and percentages were used for categorical data and numerical data were analyzed by means and standard deviation. Paired t-test was used for the analysis of the mean HDSS score difference pre- and post-treatment for both groups. Paired t-test was used for the calculation of the difference in the mean of the HDSS score between the two groups post-intervention.

## Results

We enrolled 70 patients of primary palmoplantar hyperhidrosis, 35 in each group. All patients completed the study. The mean age of the patients was 22.9 ± 3.5 years. The majority of the patients were males (74.3%) and unmarried (78.6%). Their demographics are illustrated in Table 1.

**Table 1:** Demographics and clinical data TWI: tap water iontophoresis; AC: aluminum chloride; SD: standard deviation.

Variables		Group A (TWI)	Group B (AC)	Total
Age (mean ± SD)		22.91 ± 03.23	23.05 ± 03.75	22.99 ± 03.47
Gender, n (%)	Male	25 (71.40%)	27 (77.10%)	52 (74.30%)
	Female	10 (28.60%)	08 (22.90%)	18 (25.70%)
Marital status, n (%)	Married	09 (25.70%)	06 (17.10%)	15 (21.40%)
	Unmarried	26 (74.30%)	29 (82.90%)	55(78.60%)
Education status, n (%)	Primary	00 (00.00%)	01 (02.90%)	01 (01.40%)
	Secondary	11 (31.40%)	07 (20.00%)	18 (25.70%)
	Intermediate	24 (68.60%)	23 (65.70%)	47 (67.10%)
	Graduate	00 (00.00%)	04 (11.40%)	04 (05.70%)
Family history, n (%)	Positive	18 (51.40%)	24 (68.60%)	42 (60.00%)
	Negative	17 (48.60%)	11 (31.40%)	28 (40.00%)

Comparison of mean HDSS at baseline of both group A and group B showed that no statistically significant difference was observed between them (p = 0.471). The comparison of the efficacy of treatment in terms of mean HDSS reduction for both groups is shown in Table 2.

**Table 2:** Mean HDSS score reduction HDSS: Hyperhidrosis Disease Severity Scale; TWI: tap water iontophoresis; AC: aluminum chloride; SD: standard deviation.

Variables		Group A (TWI)	Group B (AC)	P-value
Mean HDSS (mean ± SD)	Week 0	3.40 ± 0.65	3.28 ± 0.67	0.471
	Week 1	2.68 ± 0.76	2.860 ± 0.81	0.364
	Week 2	2.14 ± 0.91	2.71 ± 0.75	0.006
	Week 3	1.83 ± 0.78	2.37 ± 0.88	0.008
	Week 4	1.48 ± 0.78	2.14 ± 0.94	0.002

In group A, 34 (97%) patients had improvement in HDSS of one point or more as compared to group B (23 patients, 65.7%) (p = 0.001). Patients experienced lesser side effects in group A as compared to group B but the difference was not statistically significant (p = 0.533; Table 3).

**Table 3:** HDSS improvement and side effects HDSS: Hyperhidrosis Disease Severity Scale; TWI: tap water iontophoresis; AC: aluminum chloride; SD: standard deviation.

Variables		Group A (TWI)	Group B (AC)	P-value
HDSS improvement, n (%)	Point 0	1 (2.9%)	12 (34.5%)	
	Point 1	9 (25.7%)	8 (22.9%)	0.001
	Point 2	17 (48.6%)	12 (34.3%)	
	Point 3	8 (22.9%)	3 (8.6%)	
Side effects, n (%)	Yes	5 (14.3%)	7 (20%)	0.533
	No	30 (85.7%)	28 (80%)	

## Discussion

AC is considered the first-line treatment for primary hyperhidrosis, but it is less effective in palmoplantar hyperhidrosis [11]. In our study, we compared the efficacy and safety of TWI with AC.

Iontophoresis for palmoplantar hyperhidrosis was first used by Bouman et al. [12]. The current was applied to one hand, and the other hand was taken as control. Sweat reduction was observed in 103 (91%) out of 113 patients. Whereas, in our study, we used iontophoresis for both hands in both groups A and B. Sweat reduction was observed in 34 (97%) patients treated with TWI.

Similarly, Dahl and Glent-Madsen, in an RCT, observed 81% sweat reduction by gravimetric analysis in patients receiving maintenance therapy of iontophoresis twice weekly [13]. In our study, 97% sweat reduction was achieved in four weeks, and HDSS was used for the disease severity of the patient’s condition. It is a disease-specific tool, which provides an estimation of the impact on daily activities and treatment response over time [14].

Karakoç et al. reported an 81.2% reduction after two sessions per week for four weeks in an RCT performed on 112 palmoplantar hyperhidrosis patients. Each session of iontophoresis was of 15 minutes for each hand, and the maximum tolerable limit was used for the current application [15]. In our study, we used three sessions of iontophoresis (Monday, Wednesday, and Friday) per week for four weeks, and a 97% response was achieved.

In a randomized sham-controlled clinical trial, Kim et al. enrolled 27 patients with palmar hyperhidrosis. Iontophoresis sessions were given for 20 minutes five times a week for two weeks. Of the patients, 92.9% had clinical improvement by means of starch iodine test [16]. We performed iontophoresis for 10 minutes three times a week for four weeks, and similar results were achieved.

In a study performed by Vural et al. on the efficacy of 10 versus 20 sessions of TWI in two weeks, the findings revealed that there was no significant difference between the mean sweating intensity after the 10th and 20th sessions gravimetrically (p = 0.03) [17]. In our study, iontophoresis sessions were three sessions a week for four weeks, leading to good results and fewer side effects.

Iontophoresis with salts of aluminum alone or in a combined form with 0.01% glycopyrrolate has been used, resulting in the augmentation of sweat reduction [18,19]. We used simple tap water, which is easily available and has fewer adverse effects.

Skin irritation, such as burning and stinging, is the main adverse effect of topical AC resulting in treatment failure in many patients [20]. The same adverse effects were observed in our study in group B but they were mild and resolved spontaneously, and none of the patients left the study due to these adverse effects. Adverse effects observed with TWI were dryness, erythema, paresthesia, and vesiculation [8]. In our study, we did not observe any serious side effects except dryness, which resolved after the application of moisturizer.

Different management strategies have been proposed for the treatment of focal hyperhidrosis in various studies that include topical antiperspirants containing AC [21], iontophoresis [20], anticholinergics [22], botulinum toxin [23], microwave thermolysis [24], ultrasound therapy [25], fractional microneedle radiofrequency [26], laser [27], and surgical techniques including local excision, curettage, liposuction [28], and sympathectomy [29]. The effectiveness, safety, affordability, and simplicity of the procedure of TWI for palmoplantar hyperhidrosis still make it a valuable treatment modality. It reduces disease severity and improves the QOL, as indicated in our study.

The limitations of this study were single-center study, small sample size, and lack of follow-up. We did not follow up with the patients due to the patient load in our tertiary care hospital, and patients were coming from different cities and far-off areas leading to difficulty in follow-up.

## Conclusions

Based on our single-center study, we conclude that TWI is more effective in the treatment of palmoplantar hyperhidrosis and has lesser side effects than topical AC. It is an easily available, cost-effective, safe, and satisfying treatment modality for hyperhidrosis.

This treatment option should be suggested to patients with palmoplantar hyperhidrosis before undergoing more invasive procedures such as botulinum toxin injections and surgery. In the future, multicenter studies with a larger sample size, longer duration, and follow-up of these patients for recurrence and anticipated compensatory hyperhidrosis are highly recommended.
